# A Metagenome from a Steam Vent in Los Azufres Geothermal Field Shows an Abundance of Thermoplasmatales archaea and Bacteria from the Phyla Actinomycetota and Pseudomonadota

**DOI:** 10.3390/cimb45070370

**Published:** 2023-07-13

**Authors:** Roberto Marín-Paredes, Hermes H. Bolívar-Torres, Alberto Coronel-Gaytán, Esperanza Martínez-Romero, Luis E. Servín-Garcidueñas

**Affiliations:** 1 Laboratorio de Microbiómica, Escuela Nacional de Estudios Superiores Unidad Morelia, Universidad Nacional Autónoma de México, Morelia 58341, Mexico; roberto.marin.ibq@gmail.com (R.M.-P.); albertocoga98@gmail.com (A.C.-G.); 2 Escuela de Ciencias Biológicas, Universidad Pedagógica y Tecnológica de Colombia, Tunja 150003, Colombia; hhbolivart@unal.edu.co; 3 Centro de Ciencias Genómicas, Universidad Nacional Autónoma de México, Cuernavaca 62209, Mexico; emartine@ccg.unam.mx; 4 Laboratorio Nacional de Análisis y Síntesis Ecológica, Escuela Nacional de Estudios Superiores Unidad Morelia, Morelia 58341, Mexico

**Keywords:** *Cuniculiplasma*, *Ferrimicrobium*, thermoacidophilic Thermoplasmatales, geothermal environment

## Abstract

Los Azufres National Park is a geothermal field that has a wide number of thermal manifestations; nevertheless, the microbial communities in many of these environments remain unknown. In this study, a metagenome from a sediment sample from Los Azufres National Park was sequenced. In this metagenome, we found that the microbial diversity corresponds to bacteria (Actinomycetota, Pseudomonadota), archaea (Thermoplasmatales and *Candidatus* Micrarchaeota and *Candidatus* Parvarchaeota), eukarya (*Cyanidiaceae*), and viruses (*Fussellovirus* and Caudoviricetes). The functional annotation showed genes related to the carbon fixation pathway, sulfur metabolism, genes involved in heat and cold shock, and heavy-metal resistance. From the sediment, it was possible to recover two metagenome-assembled genomes from *Ferrimicrobium* and *Cuniculiplasma*. Our results showed that there are a large number of microorganisms in Los Azufres that deserve to be studied.

## 1. Introduction

One characteristic of many geothermal fields is the presence of steam vents, i.e., fumaroles, that consist of permanent emissions of steam and gases from the subsoil due to magmatic activity or groundwater geothermal heating [1]. Consequently, the steam has temperatures above 70 °C, wet conditions, and a concentration of minerals that form deposits as a result of the transport and evaporation of water in the walls of the caves or vents [2]. As a consequence, the walls of the vents have an important amount of minerals that provide conditions for the support of microbial life. Even though limitations of culturing thermophilic and acidophilic microorganisms exist, the use of independent culture approaches such as metagenomics helps us study in greater detail the diversity of microorganisms that inhabit the walls of the vents [3,4].

Mexico has multiple geothermal fields that have been recently explored in the Trans-Mexican Volcanic Belt, an orographic formation with high volcanic activity due to the activity of the Cocos and Rivera plates [5]. Los Azufres geothermal field is located in the state of Michoacán, Mexico, in the Trans-Mexican Volcanic Belt region, and it hosts a large number of thermal manifestations;for instance, hot springs, fumaroles, and steam vents that can reach temperatures above 90 °C and pH values below 4 [6]. The high temperatures of these sites attracted the attention of several sectors, especially the energy sector, which built a geothermal energy plant in the geothermal field. As a consequence of the development of geothermal energy generation, the researchers began to study the microorganisms that affect the different pipelines of the geothermal plant and the physicochemical characteristics of the geothermal manifestations [7,8].

The principal chemical elements found in Los Azufres are Sr, Rb, As, Na, Si, Mo, Cu, Mn, Hg, Pb, Fe, Ba, Cd, and F [9,10]. Also, chemical compounds such as sulfates, chlorides, and carbonates [6,10] were found. The major non-condensable gasses present in Los Azufres are carbon dioxide (CO_2_) and hydrogen sulfide (H_2_S). Also, there were other non-condensable gases such as ammonia (NH_3_), hydrogen (H_2_), methane (CH_4_), helium (He), nitrogen (N_2_), and argon (Ar). Carbon dioxide represents 70–90% of the total weight of non-condensable gases, while hydrogen sulfide varies between 0.2% and 13% of the total weight of non-condensable gases [11,12]. 

The microbial diversity of Los Azufres has been studied by electron microscopy or biochemical tests on corroded metals and inside pipes used for geothermal power generation [7,8,13]. Also, the microbial diversity of samples from a human-impacted site that is used as a “natural spa” for tourists was analyzed. This work was carried out by analyzing sequences of 16S rRNA genes from bacteria that were found to be related to *Acidithiobacillus* and other Proteobacteria, *Desulfurella* (Deltaproteobacteria/now Campylobacterota), Firmicutes (Bacillota), Acidobacteria (Acidobacterota), Thermotogae (Thermotogota), and Cyanobacteria (Cyanobacteriota). In a previous work, the presence of archaea was never reported [10]. 

Previously, Servín-Garcidueñas and colleagues isolated a bacterium related to *Acidocella* from an acid lake (pH 2.3) in Los Azufres [14]. Additionally, the Servín-Garcidueñas group has focused on performing metagenomic analysis to determine the microbial diversity in different areas of Los Azufres. First of all, a water sample from an acid solfatara (pH 3.6 and 65 °C) was sequenced, and there were archaea related to the order *Sulfolobales.* This archaeon was named “*Candidatus* Aramenus sulfurataquae AZ1” [15]. Also, two genomes of undescribed viruses were found belonging to the genera *Rudivirus* and *Fusellovirus* [16,17]. Subsequently, another metagenome from a yellow sediment sample around a hot spring (pH 2.8, 88.5 °C) was sequenced. In this metagenome archaea related to *Acidianus* and *Sulfolobus* were found. In addition, another genome of Ca. Aramenus [18] was obtained. In the last three years, two metagenomes have been sequenced from green sediments around a hot spring and around a fumarole. At the moment, only one genome of *Acidibrevibacterium* has been recovered from the green sediments around a fumarole [19]. 

In 2013, a metagenome was sequenced from green sediments around a steam vent, and from this metagenome, the genome of a Micrarchaeota archaea was recovered, and phylogenetic analyses of Thermoplasmatales archaea were performed [20]. The aims of this study were the assembly of genomes from metagenomic data and their analysis.

## 2. Materials and Methods

### 2.1. Sample Collection, DNA Extraction and Sequencing

**Figure 1:**
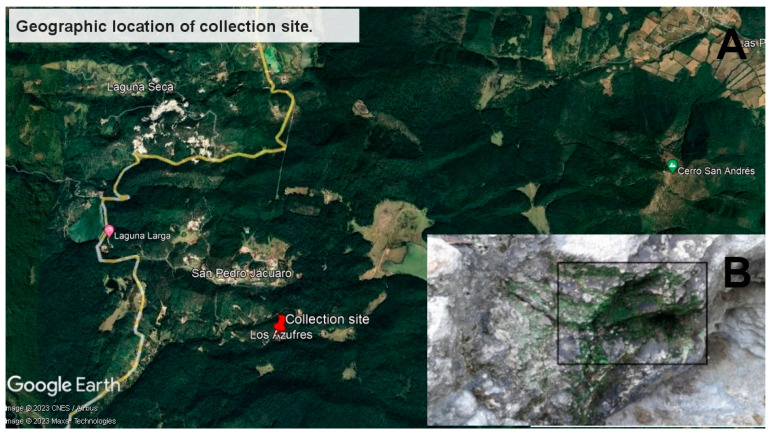
Geographic location of collection site. (**A**) Geographic location of collection site (19.78170609819753 N, −100.65805210414699 W). The Los Azufres geothermal field’s zone is shown in the image. Google-Earth-generated image. (**B**) Sample of sediment taken from the Los Azufres geothermal field. Inside the rectangle, a sample of the green sediment was obtained.

The green sediment sample was collected around a steam vent at the geothermal field of Los Azufres, Mexico, in April 2013 (Figure 1) (19.78170609819753 N, −100.65805210414699 W). The sediment sample (Figure 1) had a temperature of 67 °C and a pH of 3. DNA was isolated using the UltraClean Mega (Prep) Soil DNA Kit (MoBio Laboratories, Inc., Carlsbad, CA, USA). The sequencing was performed on an Illumina Miseq 2 × 300 bp at Macrogen, Inc., Seul, Republic of Korea.

### 2.2. Metagenome Assembly and Annotation

The reads were analyzed using FastQC v.0.11.8 [21] and filtered for quality (scores of Q ≥ 30) using Trim Galore v.0.6.7 [22]. Reads were assembled de novo with SPAdes v.3.12.0 [23] using k-mer values of 21,33,55,77,99,127. The metagenome was annotated using the server Integrated Microbial Genomes and Microbiomes (IMG/M) [24,25]. Filtered reads were used to calculate the microbial diversity of the metagenome through the software kaiju v.1.9.2 [26] with the database “nr” using translated proteins.

MetaviralSPAdes v3.15.5 [27] was used to search the metagenome for viral sequences with k-mer values of 11, 17, 23, 29, 35, 41, 47, 53, 59, 65, 71, 77, 83, 89, 95, 101, 107, 113, 119, and 127. The resulting viral sequences were verified using viralVerify v. 1.1 [27] and checked using viralComplete v. 1.0 [27] with default settings. Subsequently, these viral genomes were annotated using Prokka v.1.14.6 [28], and the resulting viral protein sequences were searched by blast to corroborate that the viral genome belonged to the virus.

### 2.3. Binning, Taxonomic Classification, and MAG’s Annotation

Metagenome-assembled genomes (MAG’s) were obtained through three different programs, such as Metabat v2.12.1 [29], Maxbin v.2.2 [30], and Concoct v.1.1.0 [31]. The results of each method were combined using DasTool v.1.1.2 [32]. The quality and taxonomic classification of each bin were checked using CheckM v.1.0.13 [33,34,35] and CAT v.5.2.3 [36], respectively. Prokka (v.1.14.6) was used to annotate each MAG [28]. Then, using COGclassifier v1.0.5, the protein sequences of each MAG were categorized into Clusters of Orthologous Groups (COGs) categories [37]. The genomic annotation of high-quality MAGs was explored in search of genes for resistance or genes involved in carbon fixation pathway, nitrogen, and sulfur metabolism. A MAG related to *Cuniculiplasma* was recovered through mapping. Bowtie2 v.2.3.4.3 (mode: very sensitive) [38] was used to map filtered reads against the genome of *Cuniculiplasma divulgatum* PM4 (LT719092.1). The mapped reads were assembled using SPAdes v.3.12.0 [23] using k-mer values of 21, 33, 55, 77, 99, and 127.

### 2.4. Phylogenomic Analysis of Thermoplasmatales archaea and Ferrimicrobium sp. AZ2-2013

Two phylogenomic trees were constructed using MAGs that have contamination less than 6.0 and completeness greater than 80%. Open reading frames (ORFs) from each bin were searched using Prokka v.1.14.6 [28]. Model organisms and type strains of Thermoplasmatales and Actinobacteria were selected from the NCBI database. The construction of the phylogenomic tree was carried out by PhyloPhlan v.0.99 [39].

### 2.5. MAG Annotation and Comparative Genomics Analysis

The MAG of *Cuniculiplasma* was annotated using the server Integrated Microbial Genomes and Microbiomes (IMG/M) [24,25]. The MAG related to *Cuniculiplasma* was compared to *Cuniculiplasma divulgatum* PM4 and *Cuniculiplasma divulgatum* S5 genomes through average nucleotide identity (ANI), average amino acid identity (AAI), and DNA-DNA hybridization (DDH) values. The values of ANI, AAI, and DDH were calculated using the ANI/AAI-Matrix calculator from the Konstantinidis Laboratory (http://enve-omics.ce.gatech.edu/g-matrix/index) (accessed on 10 September 2022) [40] and the Genome-to-Genome Distance Calculator (GGDC) (https://www.dsmz.de/services/online-tools/genome-to-genome-distance-calculator-ggdc) (accessed on 10 September 2022 ) [41]. In addition, the MAG related to *Ferrimicrobium* was compared to *Ferrimicrobium acidiphilum* DSM19497 strain T23 (GCF_000949255.1), as previously described.

## 3. Results

### 3.1. Metagenome Assembly and Annotation

**Figure 2:**
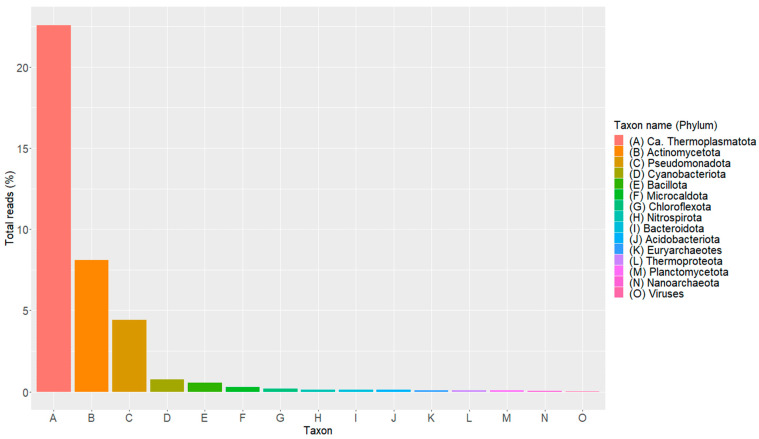
Analysis of microbial diversity of sediment sample from a steam vent of the geothermal field of Los Azufres. In the graph, each phylum is denoted by a different alphabet letter. Kaiju was used to complete the analysis of the microbial diversity.

The metagenome has 46,728,050 reads with a length between 35 and 301 bp and an average quality per read of 37 (Phred Quality Score). After the quality trimming 43,034,606 with a length between 30 and 266 and an average quality per read of 37 (Phred Quality Score) remained. The metagenome has 97,177 contigs (N50, 6617) containing 144,161,343 bp and a GC content of 55.52%. The metagenome annotation found 212,400 protein coding genes. These protein coding genes were classified using Clusters of Orthologous Groups (COGs) (Table 1). Only 37.58% of the total reads (8,087,196) were classified by Kaiju, and 62.42% of the total reads remained unclassified. According to the analysis of microbial diversity (Figure 2), the most prevalent phylum was Ca. Thermoplasmatota, which is followed by the bacterial phyla Actinomycetota, Pseudomonadota, and Cyanobacteriota.

### 3.2. Metagenome Genes Involved in Metabolic Pathway and Resistances

Genes involved in the carbon fixation pathways in prokaryotes were found. These genes correspond to the pathways of the reductive citric acid cycle, reductive acetyl-coenzyme A, dicarboxylate/hydroxybutyrate cycle, 3-hydroxypropionate bicycle, and hydroxypropionate/hydroxybutyrate cycle. However, some genes involved in the 3-hydroxypropionate bicycle and hydroxypropionate/hydroxybutyrate cycle were not found. The enzymes sulfur oxygenase/reductase (SOR) and sulfide:quinone oxidoreductase (SQR) were found. All the genes involved in dissimilatory/assimilatory nitrate reduction and assimilatory sulfate reduction were found. In addition, genes related to heat shock (HSPA1s, HSPA4, htpX, hspR) and cold shock (*cspA*) were observed. Finally, multiple genes involved in heavy metal resistance were found, such as arsenic (arsenate reductase *arsC*, arsenite transporters *arsA* and *arsB*), chromium (chromate transporter *chrA*, chromate reductase NAD(P)H dehydrogenase (quinone) *chrR*), mercury (mercuric reductase *merA*, periplasmic mercuric ion binding protein *merP*, mercuric ion transport protein merC) nickel (peptide/nickel transport system ATP-binding protein *ddpD* and *ddpF*, nickel superoxide dismutase *sodN*, peptide/nickel transport system permease protein, hydrogenase nickel incorporation protein HypA/HybF and HypB, nickel-responsive regulator *nikR*), and multi-metal (cobalt/nickel transport system ATP-binding protein *cbiO*, nickel/cobalt transporter (NiCoT) family protein *hoxN*, *nixA*, nickel/cobalt transporter (NicO) family protein *rcnA*, cobalt/nickel-transporting P-type ATPase D *ctpD*, cobalt–zinc–cadmium efflux system protein *czcD*, *zitB*, zinc and cadmium transporter *zipB*, outer membrane protein, heavy metal efflux system *czcC*, heavy metal efflux system protein *czcA*, heavy metal efflux system *czcB*). 

### 3.3. Metagenome Viral Sequences

Two viral genomes were assembled. The first viral genome (Vaz-01) belonged to *Fusellovirus* genus. Vaz-01 had a size of 14,046 bp, a GC content of 34.78%, and an identity value of 65.30% against *Sulfolobus* virus 2 (NC_005265.1.). The genome annotation of Vaz-01 revealed 21 protein coding sequences. The second viral genomes (Vaz-02) had a size of 33,639 and a GC content of 61.59%. Vaz-02 had an identity value of 69.22% against *Caudoviricetes* sp. isolate ctmKK23 (BK042626.1). Vaz-02 annotation showed 39 protein coding sequences.

### 3.4. Binning, Taxonomic Classification, and MAGs Annotation

**Figure 3:**
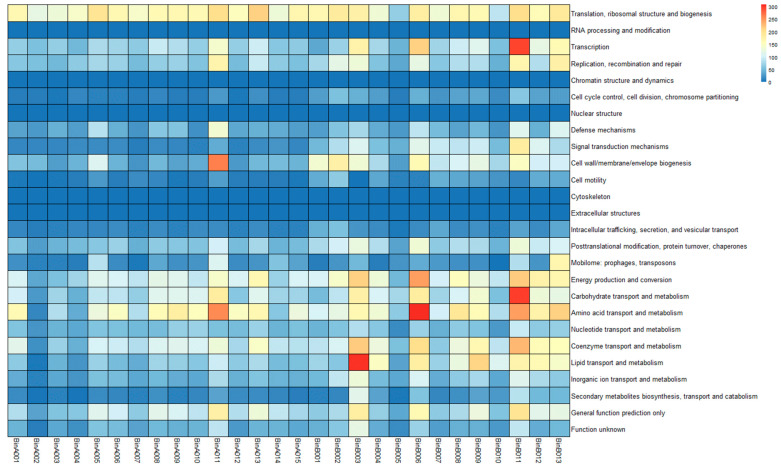
Heatmap of functional diversity representation of each MAGs. The COG category is displayed on the y axis, and the x axis displays the MAGs.

Thirty-two bins were obtained from the sediment metagenome, of which fifteen belonged to Archaea, thirteen belonged to Bacteria, and four belonged to Eukarya. Thirteen archaea were identified from the order Thermoplasmatales (“alphabet plasmas” *Cuniculiplasma* and *Ferroplasma*) and two archaea from the phylum *Candidatus* Micrarchaeota (Microcaldota) and Nanoarchaeota (Parvarchaeota order). Ten bacteria were identified related to Actinomycetota, one from the order *Acidimricrobiales* (*Ferrimicrobium* genus), another from the order *Corynebacteriales* (*Mycobacterium* genus), and eight unidentified. Furthermore, we identified three bacteria from the phylum Pseudomonadota, one from the order *Acidithiobacillales* (*Acidithiobacillus caldus*) and two unidentified. Finally, we recovered four bins of Eukarya belonging to the family *Cyanidiaceae*. The statistical information for each bin is shown in Table 2. The MAGs functional diversity analysis (Figure 3) showed that the COGs categories with a great number of genes were related to translation, ribosomal structure, and biogenesis. The analysis also revealed that the MAGs related to bacteria had a great number of genes involved in energy production and conversion, carbohydrate metabolism, amino acid metabolism, and lipid metabolism, whereas the MAGs related to archaea only had a great number of genes related to amino acid metabolism. 

Only high-quality MAGs from bacteria had genes involved in the carbon fixation pathway; BinB003 had genes related to the reductive citrate cycle. BinB002, BinB006, BinB009, and BinB012 lacked the gene for fructose-1,6-bisphosphatase II involved in the Calvin–Benson–Bassham cycle (RuBisCo). Despite the lack of this gene, they had all the genes involved in this cycle.

The enzyme sulfate sulfur oxygenase/reductase was present only in BinA002 and BinA010. All the genes involved in the assimilatory sulfate reduction pathway were present in BinB003 and BinB011. Other high-quality bacterial MAGs (BinB006, BinB009, and BinB012) did not contain the gene sulfite reductase (*SiR*), which is essential for the assimilatory sulfate reduction pathway. The entire metabolic pathway for the assimilatory nitrate reduction pathway was not present in any high-quality MAGs. The ferredoxin–nitrite reductase gene (*NirA*) was the only gene present.

Genes for heavy metal resistance were found. High-quality MAGs have the genes arsenate reductase (*arsC*) and arsenite transporters (*arsA* and *arsB*), but BinA010 and BinB004 lacked the genes for arsenic resistance. Archaeal MAGs and BinB004 lacked genes involved in mercuric resistance. Others had one of the genes for mercuric reductase (*merA*) and mercuric ion transport protein (*merC*). BinB004, BinB009, BinB011, and BinB012 had genes related to the cobalt–zinc–cadmium efflux system protein (*czcD*). Genes related to resistance to nickel and chromium were not present in any high-quality MAGs. 

There were numerous genes for heat shock proteins (*Hsp20*, *Hsp33*, and *Hsp90*) in high-quality MAGs BinB002, BinB003, BinB004, BinB011, and BinB012. Finally, only BinA010 did not have the machinery for repairing heat-induced protein damage that involves the chaperones *DnaJ*, *DnaK*, and the protein *GrpE*.

### 3.5. Phylogenomic Analysis of Thermoplasmatales archaea and Ferrimicrobium sp. AZ2-2013

**Figure 4:**
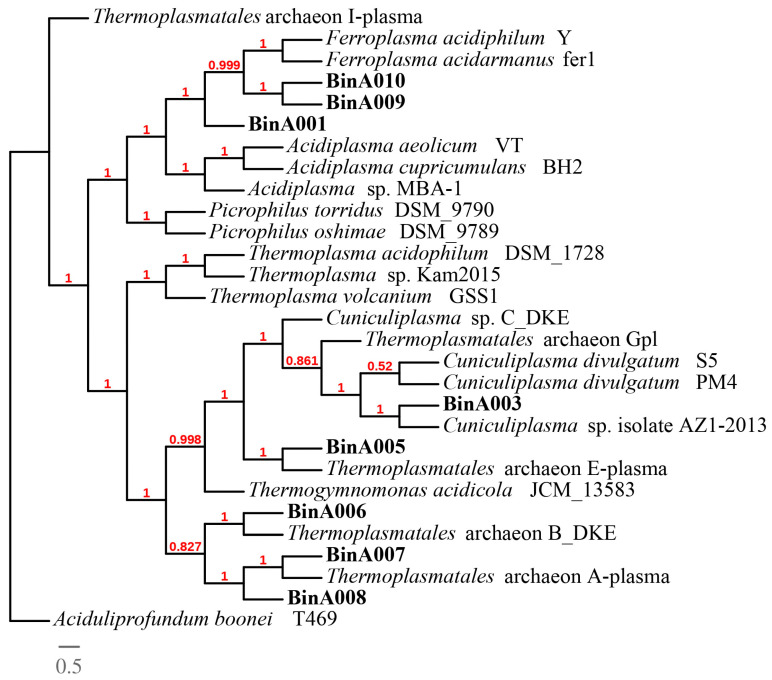
Phylogenomic tree of Thermoplasmatales archaea. The phylogenomic tree shows the predicted evolutionary relationships of genomes from the order Thermoplasmatales against genomes of Thermoplasmatales in NCBI database. MAGs recovered in this study are shown in bold letters. Aciduliprofundum boonei T469 was selected as an outgroup. Phylogenomic tree was generated using maximum likelihood model, and numbers at the branch points represent SH-like local support values. The scale bar represents the estimated number of amino acid changes per site.

**Figure 5:**
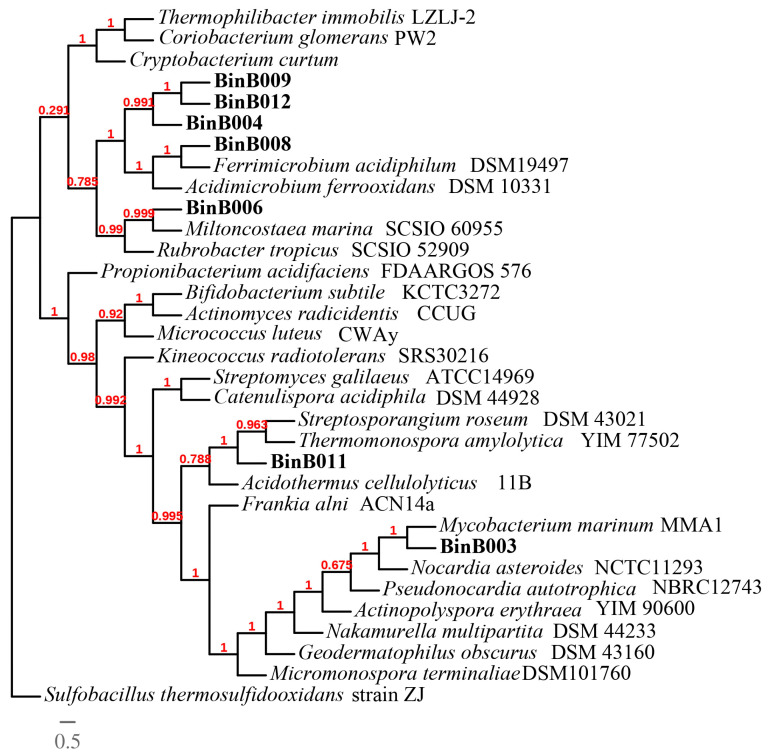
Phylogenomic tree of Actinobacteria. The phylogenomic tree shows the predicted evolutionary relationships of genomes from the phylum Actinomycetota against genomes of Actinomycetota in NCBI database. MAGs recovered in this study are shown in bold letters. *Sulfobacillus thermosulfidooxidans* strain ZJ was selected as an outgroup. Phylogenomic tree was generated using maximum likelihood model, and numbers at the branch points represent SH-like local support values. The scale bar represents the estimated number of amino acid changes per site.

The phylogenomic analysis of Thermoplasmatales archaea found in Los Azufres showed a wide diversity of these organisms (Figure 4). According to the phylogenomic analysis of Actinomycetota (Figure 5), the close relationship between BinB008 and *Ferrimicrobium acidiphilum* DSM19497, BinB003 and *Mycobacterium marinum* MMA1, and BinB006 and *Miltoncostaea marina* SCSIO 60955 was revealed. We decided to study BinB008 more deeply because it is one of the highest quality (completeness of 96.58% and a contamination of 1.38%). We propose here the next name ‘*Ferrimicrobium* sp. AZ2-2013′ to account for it.

### 3.6. MAG Annotation and Comparative Genomic Analysis

The MAG related to *Ferrimicrobium* sp. (Table 2) had 60 contigs containing 2,544,086 bp (N50 value of 60560), and it had a coverage of 6.49X and G + C content of 57.97. This MAG was called *Ferrimicrobium* sp. AZ2-2013. This MAG had values of 77.07% (ANI), 76.12% (AAI), and 19.10% (DDH) against the genome of *Ferrimicrobium acidiphilum* DSM19497 (GCF_000745905.1) and values of 77.16% (ANI), 76.06% (AAI), and 19.60% (DDH) against *Ferrimicrobium acidiphilum* DSM19497 strain T23 (GCF_000949255.1); the size of this genome was smaller compared to *F. acidiphilum* (3.08 Mb) [37]. This was the first genome of the genus *Ferrimicrobium* reported in Los Azufres. The annotation of this genome revealed many similarities between this genome and *Ferrimicrobium acidiphilum* DSM19497. There are genes related to the Calvin–Benson–Bassham cycle (RuBisCo), carboxysome, and tricarboxylic acid cycle and genes involved in iron and sulfur oxidation. Also, there are genes related to stress responses (heat shock and acid stress) and heavy metal resistance, especially arsenic and mercury.

The MAG related to *Cuniculiplasma divulgatum* had 277 contigs containing 1,858,996 bp (N50 value of 18,461), and the MAG had a coverage of 136.57X, G + C content of 37.28, and a contamination of 2.82%. This MAG was called ‘*Cuniculiplasma* sp. AZ1-2013′. This is the first genome related to Thermoplasmatales to be recovered from Los Azufres, Mexico. *Cuniculiplasma* sp. AZ1-2013 had values of 99.12% (ANI), 99.31% (AAI), and 91.50% (DDH) against the genome of *Cuniculiplasma divulgatum* PM4 (LT719092.1) and values of 98.66% (ANI), 97.88% (AAI), and 86.50% (DDH) against *Cuniculiplasma divulgatum* S5 (LT671858.1). There are genes involved in the citric acid cycle, but the enzymes 2-oxoglutarate dehydrogenase and fumarase were absent. In the synthesis of amino acids, *Cuniculiplasma* sp. AZ1-2013 lacked the genes for the synthesis of histidine, leucine, isoleucine, proline, and valine. Furthermore, there is a gene related to the heat shock protein HtpX. Notably, there are no genes related to metal resistance. 

## 4. Discussion

The microbial diversity found in sediments from Los Azufres corresponds principally to phyla Ca. Thermoplasmatota, Actinomycetota, Pseudomonadota, and Cyanobacteriota. The most prevalent microorganisms in the sediment sample from the Los Azufres geothermal area are Thermoplasmatales archaea. Only 0.060% (12, 993) (Supplementary Table S1) of the total reads from this metagenome belonged to Sulfolobales. This low abundance may be due to the type of sample. Sulfolobales archea were found in water samples from an acid solfatara [15] and in a yellow sediment sample (sulfur crystal) [18]. Thirteen MAGs associated with Thermoplasmatales were discovered by us, and phylogenomic research reveals that these archaea are very diverse. Thermoplasmatales archaea are present in acid mine drainage sites all over the world, including Parys Mountain, United Kingdom [42,43], Los Rueldos, Spain [44], and Richmond mine at Iron Mountain, United States [45,46]. Additionally, Thermoplasmatales were discovered in water samples from the Kamchatka Peninsula and Kunashir Island in Russia [47] as well as the Tenorio Volcano National Park in Costa Rica [48]. However, the temperature in Parys Mountain, UK, is between 8 and 18 °C. Thermoplasmatales were discovered to be the most prevalent microorganisms (62%) in sediment samples from Parys Mountain, UK, according to Korzhenkov et al., 2019 [42], despite the low temperatures. To further understand why Thermoplasmatales archaea are present in low temperatures, a comparative genomic analysis between the genomes discovered in Parys Mountain, UK, and genomes discovered in thermal environments is essential. 

It is important to note that bacteria from the phyla Actinomycetota and Pseudomonadota were also discovered in the Parys Mountain in the UK [42,43] and Tenorio Volcano National Park in Costa Rica [48]. Additionally, algae from the Chlorophyceae family were present in Parys Mountain [43], whereas acidophilic algae from the *Cyanidiaceae* family were found in the Los Azufres geothermal area.

Finally, we obtained viral sequences that are relevant to understanding the interactions between microbial communities. Viruses from the class Caudoviricetes have the capacity to infect both archaea and bacteria [49]. The same study mentioned that members of this class can infect cells from the order *Thermoplamatales*. However, many of the viruses in this taxonomic group remain unknown and relate to marine and anoxic environments. In the case of *Fusellovirus*, previous studies in Los Azufres reported the presence of viral sequences related to this group that infect archaea, and it is believed that this group has a key role in the gene exchange between archaea populations. As well, the presence of these viruses is common in geothermal environments [50].

According to the functional analysis, we detected that the microbial community in Los Azufres has the ability to obtain energy and resources from several sources, and bacteria have the possibility to use carbon through carbon fixation. In addition, the microbial community is capable of assimilating nitrates and sulfates. Similar adaptations have been detected in other steam vents in Mexico [3] and hot springs located in Costa Rica [48] and Malaysia [51]. Our results showed that bacteria are possible candidates for sulfate assimilation processes. Our analysis offered a vision of the versatility of the microbial community to obtain energy from several sources. The presence of genes related to heat and cold shock shows the flexibility of the microbial community to face changes in temperature. Consequently, the microbial community has been exposed to abrupt temperature changes. Furthermore, the weather in Los Azufres varies each season, reaching low temperatures. For this reason, the microbial community has developed not only strategies to live in high temperatures but also adaptations to survive in low temperatures during the winter. 

The presence of metal resistance genes is related to the presence of those metals in the environment that the microbial community inhabits; gene prediction in steam vents located in Paricutin volcano showed the presence of similar adaptations to metal resistance [3]. The heavy metals in steam vents and hot springs in Los Azufres may be due to the constant geological activity therein. For this reason, the microbial community has developed adaptations to survive and manage the metal high concentration.

In this study, we obtained the first genome related to the genus *Ferrimicrobium* from Mexico. The bacterial genus *Ferrimicrobium* was reported for the first time in the United Kingdom in a sample obtained from an abandoned sulfur mine [52,53]. There are reports of this genus in an acid river in Argentina [54], and acid mine drainage in Finland [55], Turkey [56], and Russia [57]. According to Johnson et al. [52] and Li et al. [58], *Ferrimicrobium* is able to oxidize ferrous iron and is typical of acidic environments, and it probably plays a role in the sulfur and iron biogeochemical cycle in Los Azufres due to its capacity to oxidize iron and sulfur. Furthermore, *Ferrimicrobium* sp. AZ2-2013 has genes related to metal resistance for arsenic and mercuric; heavy metal resistance was reported previously by Johnson et al. [52], but not for these metals. 

In addition, one genome of archaea related to *Cuniculiplasma divulgatum* was obtained here. *Cuniculiplasma divulgatum* was found for the first time in Spain and the United Kingdom [59]. Golyshina et al. [60] found that *Cuniculiplasma* has interactions with Ca. Micrarchaeota. Also, Golyshina et al. [61] found the presence of acidophilic algae and proposed that these algae are primary producers in these environments and *Cuniculiplasma,* being heterotrophic, could take advantage of the organic compounds produced by algae. In this metagenome, we found the presence of an acidophilic algae belonging to the genera *Cyanidiaceae* and also an archaeon related to Ca. Micrarchaeota, and it is possible that this archaeon had interactions with *Cuniculiplasma* sp. AZ1-2013. *Cuniculiplasma* sp. AZ1-2013 had high values of ANI, AAI, and DDH against *Cuniculiplasma divulgatum* PM4, indicating that *Cuniculiplasma* sp. AZ1-2013 belongs to the species *Cuniculiplasma divulgatum*. *Cuniculiplasma* sp. AZ1-2013 lacks the same genes related to the citric acid cycle and synthesis of amino acids as *Cuniculiplasma divulgatum* PM4. 

Our study expanded the knowledge of the microbial community in Los Azufres geothermal field and showed a wide variety of archaea and bacteria that were successfully assembled in MAGs. We consider it necessary to study viruses found in Los Azufres to understand their role in the ecosystem.

## Figures and Tables

**Table 1:** Protein coding sequences classified in COGs categories. COGs categories were calculated with IMG/MER.

Name	Count	Percent
Amino acid transport and metabolism	13,298	9.74%
Carbohydrate transport and metabolism	8818	6.46%
Cell cycle control, cell division, chromosome partitioning	1649	1.21%
Cell motility	1785	1.31%
Cell wall/membrane/envelope biogenesis	6417	4.70%
Chromatin structure and dynamics	120	0.09%
Coenzyme transport and metabolism	7744	5.67%
Cytoskeleton	64	0.05%
Defense mechanisms	3728	2.73%
Energy production and conversion	9578	7.02%
Extracellular structures	578	0.42%
Function unknown	5598	4.10%
General function prediction only	15,042	11.02%
Inorganic ion transport and metabolism	7063	5.17%
Intracellular trafficking, secretion, and vesicular transport	1799	1.32%
Lipid transport and metabolism	7292	5.34%
Mobilome: prophages, transposons	3466	2.54%
Nucleotide transport and metabolism	3589	2.63%
Posttranslational modification, protein turnover, chaperones	5546	4.06%
RNA processing and modification	48	0.04%
Replication, recombination and repair	6756	4.95%
Secondary metabolites biosynthesis, transport, and catabolism	4516	3.31%
Signal transduction mechanisms	4455	3.26%
Transcription	7452	5.46%
Translation, ribosomal structure, and biogenesis	10,130	7.42%

**Table 2:** Statistical information of each bin obtained from the sediment metagenome. P—phylum, C—class, O—order, G—genus, S—species. BinA—archaea, BinB—bacteria, BinE—eukarya. Quality of MAGs: high quality (completeness ≥ 90% and contamination < 5%), medium quality (completeness ≥ 70% and contamination < 10%), and low quality (completeness < 70% or contamination ≥ 10%).

Bin ID	Completeness	Contamination	Quality of MAGs	Genome Size (bp)	Number of Contigs	N50 (Contigs)	%GC	CAT/BAT Classification
BinA001	98.74	0	High quality	1,706,574	84	35,070	34.95	*Ferroplasma* (G)
BinA002	69.12	1.87	Low quality	1,420,676	702	2148	35.95	*Ca.* Parvarchaeota (P)
BinA003	82.07	0	Medium quality	1,297,165	204	13,130	37.39	*Cuniculiplasma* (S)
BinA004	81.78	1.87	Medium quality	1,253,227	101	156,283	47.39	*Ca*. Micrarchaeota (P)
BinA005	95.52	5.65	Medium quality	2,123,351	75	71,123	38.61	Thermoplasmatales archaeon “E-plasma” (S)
BinA006	95.39	4.84	High quality	1,747,204	34	88,714	44.06	Thermoplasmatales (O)
BinA007	89.47	1.61	Medium quality	1,184,382	116	15,136	43.18	Thermoplasmatales archaeon “A-plasma” (S)
BinA008	94.72	1.61	High quality	1,929,173	66	46,843	42.25	Thermoplasmatales (O)
BinA009	97.65	5.69	Medium quality	1,716,143	40	79,707	37.91	*Ferroplasma* (G)
BinA010	94.05	3.25	High quality	1,556,444	61	42,667	39.16	*Ferroplasma* (G)
BinA011	80.14	28.83	Low quality	3,842,114	370	14,845	40.9	Thermoplasmatales (O)
BinA012	72.92	20.33	Low quality	1,642,787	523	4064	44.62	Thermoplasmatales archaeon “A-plasma” (S)
BinA013	96.37	19.35	Low quality	2,229,358	143	64,732	44.7	Thermoplasmatales archaeon “I-plasma” (S)
BinA014	71.77	17.34	Low quality	1,586,617	201	14,693	41.52	Thermoplasmatales (O))
BinA015	74.69	24.69	Low quality	1,450,377	162	15,441	43.6	Thermoplasmatales (O)
BinB001	93.97	0	High quality	1,754,364	20	114,024	66.67	Gammaproteobacteria (C)
BinB002	99.38	0	High quality	2,395,071	53	80,710	62.41	Pseudomonadota (P)
BinB003	99.55	0.45	High quality	4,091,439	176	47,256	66.49	*Actinomycetales* (O)
BinB004	92.74	0.85	High quality	2,246,041	483	6521	58.95	Actinomycetota (P)
BinB005	39.56	0.85	Low quality	1,299,422	822	1563	66.42	Bacteria
BinB006	97.86	1.14	High quality	3,765,924	74	128,327	70.35	Actinomycetota(P)
BinB007	62.65	1.3	Low quality	1,844,347	418	4609	65.59	Gammaproteobacteria (C)
BinB008	96.58	1.38	High quality	2,544,086	60	60,560	57.97	*Ferrimicrobium* (S)
BinB009	94.79	2.91	High quality	2,895,140	349	10,670	73.1	Actinomycetota (P)
BinB010	51.91	3.23	Low quality	1,990,973	1119	1860	73.16	Bacteria
BinB011	97.59	3.48	High quality	5,183,776	320	24,927	71.89	*Actinomycetales* (O)
BinB012	94.87	4.7	High quality	2,730,528	96	50,090	73.93	Actinomycetota (P)
BinB013	94.02	8.93	Medium quality	3,691,086	559	8930	68.55	Bacteria
BinE001	NA	NA	NA	162,145	15	89,086	37.92	*Cyanidiaceae* (F)
BinE002	NA	NA	NA	11,989,646	192	113,036	53.45	*Cyanidiaceae* (F)
BinE003	NA	NA	NA	113,433	6	78,601	28.14	*Cyanidiaceae* (F)
BinE004	NA	NA	NA	142,617	5	72,671	28.44	*Cyanidiaceae* (F)

## Data Availability

Raw sequencing data are available in the NCBI Sequence Read Archive (SRA) under accession number SRR18192416. The MAGs for “*Cuniculiplasma* sp. AZ1-2013” and “*Ferrimicrobium* sp. AZ2-2013” are available in GenBank under accession numbers JALBYY000000000 and JALCZJ000000000, respectively. The metagenomes and “*Cuniculiplasma* sp. AZ1-2013” functional annotations are available from the JGI Genome Portal under accession numbers 264294 and 212543, respectively.

## Supplementary Materials

The following supporting information can be downloaded at: https://www.mdpi.com/article/10.3390/cimb45070370/s1, Table S1: Microbial diversity of sediment sample at the taxonomic level of order.
